# Improving the Recording of Diagnoses in Primary Care with Team Incentives: A Controlled Longitudinal Follow-Up Study

**DOI:** 10.1155/2018/4606710

**Published:** 2018-02-20

**Authors:** Tuomo Lehtovuori, Timo Kauppila, Jouko Kallio, Anna M. Heikkinen, Marko Raina, Lasse Suominen, Reijo Sund

**Affiliations:** ^1^City of Espoo, Administration of Primary Care, Espoo, Finland; ^2^Department of General Practice and Primary Healthcare, University of Helsinki, P.O. Box 20, Tukholmankatu 8 B, 00014 Helsinki, Finland; ^3^City of Vantaa, Administration of Primary Care, Vantaa, Finland; ^4^Department of Oral and Maxillofacial Diseases, Institute of Dentistry, University of Helsinki, P.O. Box 41, 00014 Helsinki, Finland; ^5^Centre for Research Methods, Department of Social Research, University of Helsinki, P.O. Box 18, Unioninkatu 35, 00014 Helsinki, Finland

## Abstract

**Introduction:**

We studied whether primary care teams respond to financial group bonuses by improving the recording of diagnoses, whether this intervention leads to diagnoses reflecting the anticipated distribution of diseases, and how the recording of a significant chronic disease, diabetes, alters after the application of these bonuses.

**Methods:**

We performed an observational register-based retrospective quasi-experimental follow-up study with before-and-after setting and two control groups in primary healthcare of a Finnish town. We studied the rate of recorded diagnoses in visits to general practitioners with interrupted time series analysis. The distribution of these diagnoses was also recorded.

**Results:**

After group bonuses, the rate of recording diagnoses increased by 17.9% (95% CI: 13.6–22.3) but not in either of the controls (−2.0 to −0.3%). The increase in the rate of recorded diagnoses in the care teams varied between 14.9% (4.7–25.2) and 33.7% (26.6–41.3). The distribution of recorded diagnoses resembled the respective distribution of diagnoses in the former studies of diagnoses made in primary care. The rate of recorded diagnoses of diabetes did not increase just after the intervention.

**Conclusions:**

In primary care, the completeness of diagnosis recording can be, to varying degrees, influenced by group bonuses without guarantee that recording of clinically significant chronic diseases is improved.

## 1. Introduction

Financial incentives can be defined as “all the rewards agents (physicians or the physician organization) receive conditional on their measured (explicitly or implicitly) performance or behavior” [1]. The system by which physicians are paid may affect their professional practice and decision-making [2, 3]. Therefore, differently tailored payment systems for general practitioners (GPs) have been used to achieve the desired policy objectives, such as improving the quality of care or recruitment to underserved areas [2, 3]. There is ample recent research about how pay-for-performance systems to GPs alter their clinical activities [4–10]. They can also be used to reduce inequalities in the delivery of clinical care related to area deprivation [4]. There are quite autonomous actors other than physicians, such as nurses who specialize in the treatment of diabetes [11–14], who may influence the clinical practice of their organization dramatically and even improve the quality of care [11, 14]. Therefore, disciplines other than GPs might well be considered as targets for financial incentives.

Insufficient recording of diagnoses may hamper planning of healthcare and adequate allocation and management of resources [15] and improving the recording of chronic diseases might theoretically serve as one of the first targets in improving the quality of care [15, 16]. Therefore, the administration of the primary healthcare of Espoo City considered the recording of diagnoses in only 40–60% of doctor visits to be insufficient [17]. Former studies suggested that financial incentives to GPs increased the recording of diagnoses [5, 7] and a preliminary analysis suggested that group bonuses could do the same [17]. With financial group bonuses to care teams, the administration of Espoo primary care wanted to improve the recording of diagnoses, especially the recording of diabetes diagnoses. Diabetes is known to require considerable care and causes a lot of costs [16, 18–20].

The main aim of this present study was to quantify the extent of the effect of group bonuses on improvement in the level of marking diagnoses in the patient chart system. We examined whether all teams responded equally to this intervention. The administration was also concerned about the adequacy of the marked diagnoses because any diagnosis recording, even inadequate, produced financial rewards. Therefore, we investigated the range of diagnoses which were recorded to find out whether the present financial intervention produced data which reflected the distribution of diagnoses in real clinical life in primary care and thus provided valid data about public health. As an example of a significant chronic public disease, the recording of diabetes [16, 18–20] was monitored to find out whether this disease was recorded more frequently after the present intervention. To provide impression about costs group bonus incurred for Espoo primary care, the percentage of staff receiving bonuses was recorded, as well as the mean bonus per year for one staff member.

## 2. Methods

### 2.1. Design and Setting

The present research is a retrospective longitudinal quasi-experimental study with a before-and-after design in 5 primary healthcare areas, with 3–6 care teams (cells) each, in Espoo (230,000 and 254,000 inhabitants in 2006 and 2012, resp.). Altogether, the number of cells was 23. There were 6–8 doctors and 6–8 nurses in each cell. The precise number of doctors and nurses varied slightly over the study period. More detailed information about the functions and frequency of use in Espoo primary care at the time of this study was described previously [17].

No ethical approval was required because this study was made directly on a computer from the patient register without identifying the patients (https://rekisteritutkimus.wordpress.com/luvat-ja-tietosuoja/). The register keeper (the health authorities of Espoo and Vantaa, 23.8.2016) granted permission to carry out the study. The report generator automatically allowed following the monthly number of recorded diagnoses for each individual doctor and therefore also by each individual cell.

As control data, we had the respective data on the recording of monthly diagnoses from two different primary care units where there was a similar cell structure but no team incentives were applied. Dental primary care of Espoo was chosen because both somatic and dental primary care are under the same administration, and we wanted to see whether desired practices disperse to other parts of the same primary care system without actual intervention. The primary care of Vantaa city was chosen because it resembles Espoo in its location and number of inhabitants (about 200,000 inhabitants, located neighboring Helsinki, the capital of Finland).

### 2.2. Intervention

The chosen intervention to increase the number of recorded diagnoses was to pay bonuses to all members of the care teams who met their target. The administration of Espoo primary care defined the focus areas and their goal levels at the start of 2005 and improvement in recorded diagnoses on the patient charts was chosen as the main goal. Before 1.3.2005, no group bonuses to the cells were delivered. To commit the staff to the change in function, a multidisciplinary team contract was signed with the members of the cells. The contract defined the rules and approaches to the functions of the cells. The team contracts were signed by all of the five service areas for the period 1.3.2005–30.5.2005, which was considered to be the time of the start of the intervention. After signing this contract, the cells were able to aim for group bonuses. This meant that, to get a group bonus, a care team had to take care that diagnoses were recorded in more than 75% of all doctor visits of that team.

### 2.3. Primary and Secondary Outcomes

The proportion of monthly doctor visits having recorded diagnoses was selected as the main measure to study the effect of implementing group bonuses. Diagnoses were recorded with the ICD-10 system by the doctors.

To study the effects of group bonuses at the individual cell level, the team-based monthly percentage of visits having marked diagnoses was derived from electrical patient charts from each care team in Espoo primary care. We calculated the monthly variation of these percentages in each cell during the year 2006 when the intervention was already fully functioning. The variation between care teams was analyzed using these aggregated cell-level percentages. To establish whether the team incentive system had altered the recording of diagnoses of chronic diseases, the monthly numbers of diabetes diagnoses (ICD-10 codes E10 and E11) were also studied in Espoo health center. Simultaneously, all diagnoses recorded during 2006 were also gathered.

The costs of group bonuses were obtained from years 2005 and 2006 from the payroll system of the social and health bureau of Espoo. The percentage of staff receiving bonuses and annual bonus per staff member were recorded.

### 2.4. Data Extraction

The effect of the intervention on the proportion of monthly doctor visits with recorded diagnoses was continuously monitored for a two-year time period before intervention and 1.5 years after it. The data about the recorded diagnoses was specifically derived from the electronic Effica patient chart system (Tieto Ltd., Helsinki, Finland) from which the data were reliably obtainable from 1.5.2003. The control data from Espoo dental care were similarly obtainable from 1.5.2003. The control data from Vantaa health center were obtained from the graphic Finstar patient chart system (Logica Ltd., Helsinki, Finland). The Effica and Finstar patient chart systems have a similar setting in the site where the diagnosis is supposed to be marked. Writing the three first symbols (letters when using directly a diagnosis or a letter and two numbers when using directly an ICD-10 code) opens automatically a list of all putative diagnoses with that symbol combination. Then, the GP or dentist can choose by double-clicking the desired diagnosis from these options.

### 2.5. Statistical Analysis

To study the effect of intervention on the frequency of marking diagnoses in GP visits, we used interrupted time series (ITS) ARIMA model [21] to compare the percentages of monthly doctor visits with recorded diagnoses before and after intervention. The same testing methods were used when these proportions were compared with the control units (Espoo dental healthcare and Vantaa primary care).

The variations between care teams in Espoo primary care were analyzed using the aggregated cell-level percentages in 2006 and performing One-Way Repeated Measures- (RM-) ANOVA on ranks with suitable corrections (Tukey) for multiple comparisons or when following the development of an individual care team as a function of time.

To study whether the team incentive system altered the recording of diagnoses of chronic diseases, the monthly number of diabetes diagnoses (ICD-10 codes E10.*∗∗* and E11.*∗∗*) was also analyzed with One-Way Repeated Measures Analysis of Variance followed by Bonferroni correction for multiple comparisons.

## 3. Results

**Figure 1:**
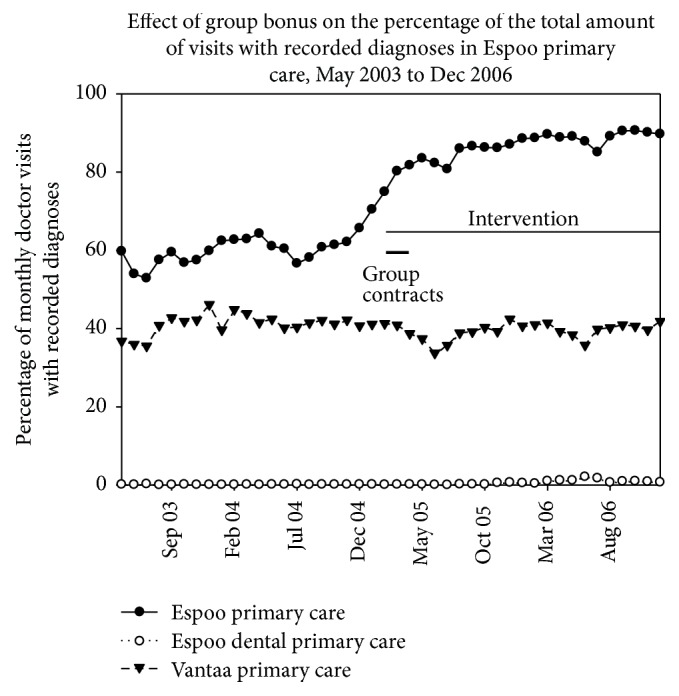
Effect of group bonus on the percentage of total amounts of visits to doctors with recorded diagnoses in Espoo primary care and controls. Follow-up time is May 2003 to Dec 2006.

Based on the ITS analyses, the proportion of recorded diagnoses in Espoo primary care increased on average by 17.9% units (95% CI: 13.6–22.3; *P* < 0.001, ITS analysis) from 59–70% up to 90% after applying group bonuses, while there was no increase in the controls (Vantaa primary care −2.0% [−4.1–0.1] and Espoo dental care −0.3% [−1–0.4]; see Figure 1).

**Figure 2:**
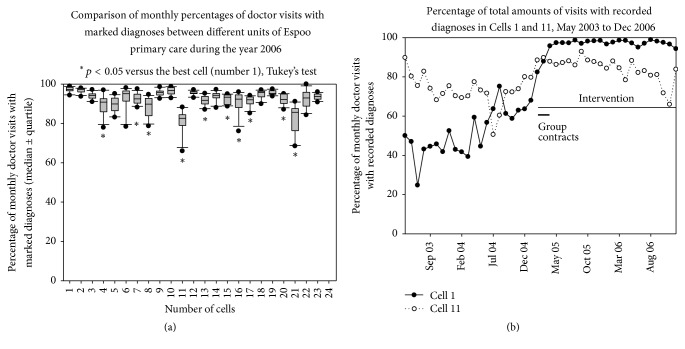
(a) Comparison of monthly percentages of visits to GPs with recorded diagnoses between different units of Espoo primary care during the year 2006. Median and 25% and 75% quartiles are presented with a box plot, 10 and 90% limits are presented with brackets, and the lowest and highest values are presented with dots. (b) Effect of group bonus on the percentage of total amounts of visits to GPs with recorded diagnoses in Cells 1 and 11. Follow-up time is May 2003 to Dec 2006.

During intervention in 2006, there was still considerable variation (Figure 2(a)) between different cells of Espoo health center in the recording of diagnoses during monthly doctor visits (*P* < 0.001, Figure 2(a)). Despite this difference in the level of recorded diagnoses, all the cells improved their performance after intervention, and in the most active cell, Cell 1, this improvement was 33.7% (26.6–41.3; *P* < 0.001, ITS analysis), while in the least active unit, Cell 11, it was less than half of that (14.9% [4.7–25.2; *P* = 0.007], Figure 2(b)).

**Figure 3:**
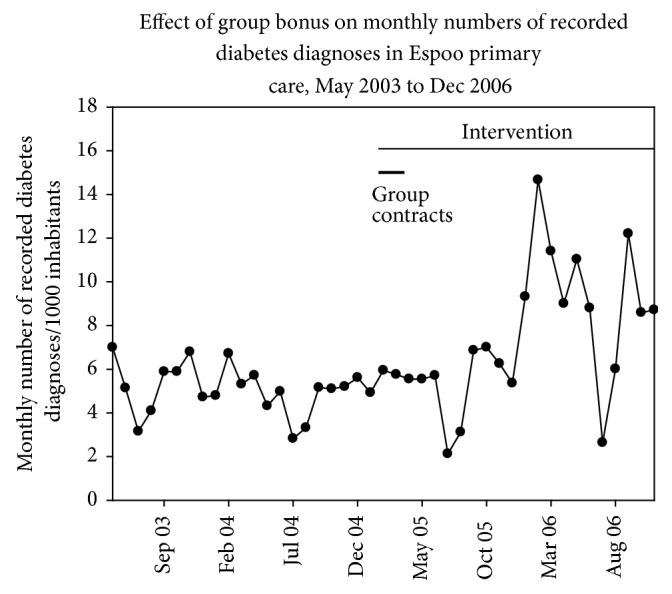
Effect of group bonus on monthly numbers of recorded diabetes diagnoses in visits to GPs. Follow-up time is May 2003 to Dec 2006.

Altogether, 2,984 different ICD-10 diagnoses were assessed during the year 2006 by Espoo GPs. The total number of assessed diagnoses was 73,912. The distribution of the most used diagnoses in 2006 is described in Table 1. Most of the visits concerned mild respiratory infections, elevated blood pressure, low back pain, type II diabetes, and infectious gastroenteritis. The median rate of monthly doctor visits, in which diabetes diagnoses were recorded, doubled after intervention (*P* < 0.001, RM-ANOVA, median: 208; IQR: 96.8) in 2006 when compared with preceding years (2003 [108; 71.5], 2004 [117; 29.5], or 2005 [134; 35]). According to ITS analysis, there was, however, no statistically significant intervention effect (1% [95% CI: −2.3–4.2]; Figure 3). During the same follow-up period, the number of all monthly doctor visits varied between 21,506 (95% CI: 20,072–22,941 in 2004) and 22,243 (20,657–23,827 in 2005) visits (*P* = 0.24, RM-ANOVA).

The annual bonus per staff member proved to be about 700 euros (Table 2). The percentage of staff reaching the group bonus was about 50% of the total staff.

## 4. Discussion

Multidisciplinary rewarding with financial incentives, for example, group bonus with team contracts, improved the rate of marking of diagnoses in the patient charts by about 18%. This improvement was not, however, observed with a chronic disease diagnosis, diabetes. There were still considerable variations between the cells in the levels of recording the diagnoses after applying group bonuses. All obtained diagnoses accurately reflected the anticipated distribution of diseases in Finnish primary care.

In accordance with the present findings regarding group bonuses, former studies suggested that financial incentives to GPs increased the recording of diagnoses and thereby the recording of diseases [5, 7]. Various and numerous administrational factors and management problems [17] were supposed to create hindrances to the proper recording of patient data, but group bonuses seemed to overcome their effects. The present data and some recent studies [6, 22, 23] suggest that financial incentives may be used to alter the behavior of GPs towards improving the quality of care. At least some aspects of quality, for example, indirect indicators of the quality of care, may show an improvement with pay-for-performance systems while patient outcomes may not necessarily do so [6, 10]. Taken together with the former results, our data support the view that financial incentives clearly modify system centered indicators of quality of care [24–26].

The present intervention was aimed at ensuring that the diagnoses were marked for projects directed towards improving the management of chronic illnesses in Espoo primary care [17]. Group bonuses were not, however, necessarily the cause of the observed increase in the recording of an important public health problem, namely, diabetes. An alternative explanation is that group bonuses were effective in increasing the recording of diabetes but that these diagnoses occurred so seldom and the change was so slow that ITS analysis failed to detect it. Nevertheless, it is not confirmed whether group bonuses are effective primers in primary care interventions which are directed towards chronic public diseases.

We cannot answer the question of why group bonuses were more effective in enhancing the recording of diagnoses in some teams and less effective in others with the present data. Roughly 60 euros extra per month is a considerable addition to salary for other members of the team than GPs. To offset “free riders” and to avoid dilution of the incentive, the teams must be relatively small [27], which fitted well with the present care teams of 16 persons or less. This relatively small size of the care teams (e.g., cells) could have created group pressure motivating the whole team to improve performance. Money is an essential factor, but other mechanisms, such as group pressure, may play a role in financial incentive-based interventions. To support this, partial withdrawal of financial incentives did not lead to a deterioration of results obtained with a GP-based pay-for-performance intervention [9] and the mean team-based rate of recording diagnoses decreased only a little after total withdrawal of group bonuses [17].

In the present study, most of the visits (Table 1) concerned mild respiratory infections, elevated blood pressure, low back pain, and type II diabetes. There was no extra education in the intervention group for better registration of the diagnoses. Pärnänen et al. [28] reported that upper respiratory infections and otitis media, hypertension, musculoskeletal pains, and diabetes were the most common reasons to visit a GP in a Finnish health center. In Denmark, the most common reasons to visit a GP were reported to be musculoskeletal, respiratory, and skin related diagnoses followed by psychological, circulatory, and metabolic disorders when the ICPC system was used [29]. Thus, the diagnoses recorded due to the present financial intervention would most likely seem to reflect real clinical life in Finnish primary care and the present intervention may provide reliable data about public health.

The main strength of the present study was the completeness of the data. The computerized patient chart system reached every single doctor in the public primary care in Espoo and Vantaa and every public primary care dentist in Espoo. The accuracy of all the diagnoses cannot be guaranteed in the present experiment. There are differences in how individual GPs code their diagnoses. However, the data were so large that differences in coding between different GPs are likely to vanish in random deviation.

## 5. Limitations

The present study does not provide clear information about whether group bonuses are applicable in other parts of healthcare. Neither does it give new answers to the key question of whether the use of these financial incentives can improve direct measures in public health or patient centered indicators of care in primary care. Qualitative studies or comparing group bonuses with other types of incentive-based interventions should be performed to gain information about how group incentives work and why there is so much variability in the effect of group bonuses.

Group bonus may alter the behavior of primary care teams. However, it is not necessarily an effective intervention for the treatment of chronic diseases and its efficacy in care teams is not very constant. The administration of healthcare must carefully consider which behavior is rewarded before intervening in clinical activity with group bonuses.

## 6. Conclusion

Group bonuses improve the completeness of diagnosis coding in primary care but the variability of this effect on the different primary care groups is substantial. They do not guarantee better recording of chronic diseases. All obtained diagnoses accurately reflected the anticipated distribution of diseases.

## Figures and Tables

**Table 1:** The distribution of the most used diagnoses in primary care doctor visits in 2006.

ICD-10	Diagnosis	Number of patients	%
J06.9	Acute upper respiratory airway infection, nonspecific	9680	13,1
I10	Primary hypertension	2708	3,7
M54.5	Low back pain	2202	3
J01.0	Acute maxillary sinusitis	2061	2,8
E11	Diabetes, type II	1686	2,3
A09	Diarrhea and gastroenteritis, presumed infectious origin	1290	1,7
H66.9	Acute otitis media, nonspecific	1195	1,6
J20.9	Acute bronchitis, nonspecific	1184	1,6
M54	Back pain	988	1,3
G44.2	Tension headache	940	1,3
H66.0	Acute otitis media, purulent	854	1,2
M54.4	Low back pain with sciatica	647	0,9
S93.4	Ankle sprain	584	0,8
H10.3	Acute conjunctivitis, nonspecific	565	0,8
J03.9	Acute tonsillitis, nonspecific	540	0,7
N30.0	Acute cystitis	532	0,7
H10.0	Conjunctivitis, purulent	514	0,7
R10.4	Abdominal pain, nonspecific	512	0,7
H65.0	Acute otitis media, serous	500	0,7
J04.0	Acute laryngitis	487	0,7
J02.9	Acute pharyngitis, nonspecific	466	0,6
E78.01	Hypercholesterolaemia, primary	452	0,6
F43.0	Acute stress reaction	394	0,5
R10.3	Lower abdominal pain	388	0,5
M77.1	Lateral epicondylitis	383	0,5
I48	Atrial fibrillation	379	0,5
F32.9	Depression, nonspecific	370	0,5
J45	Bronchial asthma	367	0,5
I25.1	Coronary artery disease	352	0,5
R50.9	Fever, nonspecific	345	0,5
M75.1	Rotator cuff syndrome	338	0,5
J01.9	Acute sinusitis, nonspecific	319	0,4
F32	Depression	304	0,4
R53	Fatigue	291	0,4
E10	Diabetes, type I	289	0,4
F51.0	Insomnia, nonorganic	277	0,4
M53.0	Cervicocranial syndrome	272	0,4
R05	Cough	271	0,4
R51.80	Sleep disorder, specific nonorganic	264	0,4

**Table 2:** Percentage of staff receiving group bonus, number of staff, and mean size of bonus in 2005 and 2006.

Health service areas	Percentage of staff receiving bonus		Mean annual bonus/person (€)	
	2005	2006	2005	2006
Area 1	44,0	50,0	601,77	716,85
Area 2	16,0	49,0	205,15	670,97
Area 3	36,0	49,0	528,77	762,57
Area 4	26,0	41,5	347,85	592,45
Area 5	36,0	55,7	502,21	768,75

## Abbreviations

GP: General practitioner
ITS: Interrupted time series.
